# Interchangeability of right ventricular longitudinal shortening fraction assessed by transthoracic and transoesophageal echocardiography in the perioperative setting: A prospective study

**DOI:** 10.3389/fcvm.2022.1074956

**Published:** 2022-12-23

**Authors:** Christophe Beyls, Pierre Huette, Paul Vangreveninge, Florent Leviel, Camille Daumin, BenAmmar Ammar, Gilles Touati, Bouzerar Roger, Thierry Caus, Hervé Dupont, Osama Abou-Arab, Diouf Momar, Yazine Mahjoub

**Affiliations:** ^1^Department of Anesthesiology and Critical Care Medicine, Amiens University Hospital, Amiens, France; ^2^UR UPJV 7518 SSPC (Simplification of Care of Complex Surgical Patients) Research Unit, University of Picardie Jules Verne, Amiens, France; ^3^Department of Cardiac Surgery, Amiens University Hospital, Amiens, France; ^4^Department of Biophysics and Image Processing, Amiens University Hospital, Amiens, France; ^5^Department of Biostatistics, Amiens University Hospital, Amiens, France

**Keywords:** right ventricular shortening fraction, speckle tracking, interchangeability, right ventricle, tricuspid

## Abstract

**Background:**

Conventional transthoracic (TTE) and transoesophageal echocardiography (TEE) parameters assessing right ventricle (RV) systolic function are daily used assuming their clinical interchangeability. RV longitudinal shortening fraction (RV-LSF) is a two-dimensional speckle tracking parameter used to assess RV systolic function. RV-LSF is based on tricuspid annular displacement analysis and could be measured with TTE or TEE.

**Objective:**

The aim of the study was to determine if RV-LSF_TTE_ and RV-LSF_TEE_ measurements were interchangeable in the perioperative setting.

**Methods:**

Prospective perioperative TTE and TEE echocardiography were performed under general anesthesia during scheduled cardiac surgery in 90 patients. RV-LSF was measured by semi-automatic software. Comparisons were performed using Pearson correlation and Bland-Altman plots. RV-LSF clinical agreement was determined as a range of −5 to 5%.

**Results:**

Of the 114 patients who met the inclusion criteria, 90 were included. The mean preoperative RV-LSF_TTE_ was 20.4 ± 4.3 and 21.1 ± 4.1% for RV-LSF_TEE._ The agreement between RV-LSF measurements was excellent, with a bias at −0.61 and limits of agreement of −4.18 to 2.97 %. All measurements fell within the determined clinical agreement interval in the Bland-Altman plot. Linear regression analysis showed a high correlation between RV-LSF_TTE_ and RV-LSF_TEE_ measurement (r = 0.9; confidence interval [CI] 95%: [0.87–0.94], *p* < 0.001).

**Conclusion:**

RV-LSF_TTE_ and RV-LSF_TEE_ measurements are interchangeable, allowing RV-LSF to be a helpful parameter for assessing perioperative changes in RV systolic function.

**NCT:**

NCT05404737. https://www.clinicaltrials.gov/ct2/show/NCT05404737.

## Introduction

Echocardiography is a non-invasive, simple, and helpful technique in patients undergoing interventional cardiology procedures, cardiac surgery, high-risk non-cardiac surgery, and for diagnosing intra- or post-operative complications (1). Two-dimensional transthoracic echocardiography (TTE) and transoesophageal echocardiography (TEE) are routinely used to qualitatively and quantitatively evaluate the structure and function of the heart (2). TTE and TEE echocardiographic assessment of the right ventricular (RV) systolic function is challenging and requires a multiparametric approach that combines conventional parameters, such as tricuspid annular plane displacement (TAPSE), RV fractional area change (RV-FAC), and advanced speckle tracking parameters (3).

In interventional cardiology procedures, TEE is used to guide device placement and assess the periprocedural changes of RV systolic function (4). During cardiac surgery, intraoperative TEE is crucial in assisting surgical decision-making. It is also a helpful monitoring tool for providing an immediate point-of-care assessment of RV systolic function, especially with TAPSE and RV-FAC parameters (1, 3, 5). In the intensive care unit, RV systolic function is routinely assessed using conventional parameters measured by transthoracic echocardiography. This evaluation is crucial for RV failure diagnosis, global hemodynamic management, and ventilator parameters setting (5). In daily practice, RV systolic parameters measured by TTE and by TTE are often used interchangeably and assumed clinically equivalent, even if their measurement must be done with caution (6). Actually, the values of conventional RV-systolic function parameters obtained in TTE and TEE were not comparable due to a large variability, a poor correlation (8), and an underestimation with TEE (7). For some authors, RV strain parameters and 3D RV ejection fraction (3D-RVEF) should be used to avoid the variability and angle dependency of the conventional RV systolic parameters (6). However, the measurement of RV strain parameters and 3D-RVEF required high image quality and specific probes, thus limiting their use in clinical routine.

The right ventricle longitudinal shortening fraction (RV-LSF) is a two-dimensional speckle tracking echocardiography (2D-STE) parameter based on the longitudinal tricuspid annular displacement (TAD) that assesses the global RV systolic function (9). RV-LSF is a semi-automatic, angle-independent, and accurate 2D-STE parameter for assessing RV systolic dysfunction in several clinical settings (10, 11). Besides, RV-LSF is a fast and reproducible post-processing 2D-STE parameter less dependent on image quality (12) and loading conditions (13) than RV strain parameters. RV-LSF combines the longitudinal displacement of the lateral (TAD_lat_) and septal (TAD_sep_) portion of the tricuspid ring toward the RV apex. RV-LSF can be measured by TTE or TEE (14). In TTE, RV-LSF is more correlated to the RV ejection fraction, evaluated in magnetic resonance imaging (9) or three-dimensional echocardiography (12), than conventional and strain parameters. Besides, RV-LSF is more accurate for identifying patients with RV dysfunction (10).

However, to our knowledge, RV-LSF values measured by TTE and TEE have not been compared. Mainly, there are no data assessing the interchangeability of RV-LSF_TTE_ and RV-LSF_TEE_. In TEE, several factors could affect RV-LSF measurement and, therefore, its interchangeability: TEE view is foreshortened and does not fully display the apical portion of the RV, which is crucial for ROI placement. Besides, the dynamic and non-planarity of the tricuspid annulus could also affect the measurement of TAD_lat_ and TAD_sep._

The first aim of the study was to determine whether RV-LSF_TTE_ and RV-LSF_TEE_ measurements could be considered interchangeable in the perioperative setting. The second aim was to study the interchangeability of the two components of RV-LSF (TAD_lat_ and TAD_sep_).

## Methods

### Study population

This prospective interventional study was conducted at Amiens university hospital (Amiens, France) between August 2021 and April 2022. We prospectively included all adult patients (>18 years old) hospitalized for a scheduled cardiac surgery under cardiopulmonary bypass that required intraoperative TEE. Exclusion criteria were patients with a contraindication to TEE performance (gastric or esophageal pathology), a poor echogenicity on TEE, a TTE not allowing evaluation of RV-LSF, and patients with a rapid supraventricular rhythm disorder at the time of TEE and TTE.

### Ethics

This is a single-center, prospective and interventional study of patients hospitalized at Amiens University Hospital for scheduled cardiac surgery under cardiopulmonary bypass (CPB). The study was approved by the Amiens University Hospital IRB (CHU–Place V. Pauchet, 80054 AMIENS Cedex) and by an institutional ethics committee (Comité de Protection des Personnes Ile de France VIII, ID-RCB 2021_A000908-33). Oral and written information was provided to the patients.

### Echocardiography procedure

Echocardiography images were obtained using high-quality commercially available probes (S5-1 for TTE, X7-2T for TEE, Philips Healthcare) and ultrasound systems (CX 50, Philips Healthcare). To assess the interchangeability of RV-LSF, TTE and TEE exams, respectively, were performed in patients under general anesthesia immediately after induction of anesthesia, oral intubation, and muscle blockade. The procedures for general anesthesia and mechanical ventilation were standardized for all patients. The TEE and TTE echocardiography protocols followed the American Society of Echocardiography and the European Society of Cardiology recommendations for assessing RV systolic function (3, 15).

### RV-LSF measurement

**Figure 1:**
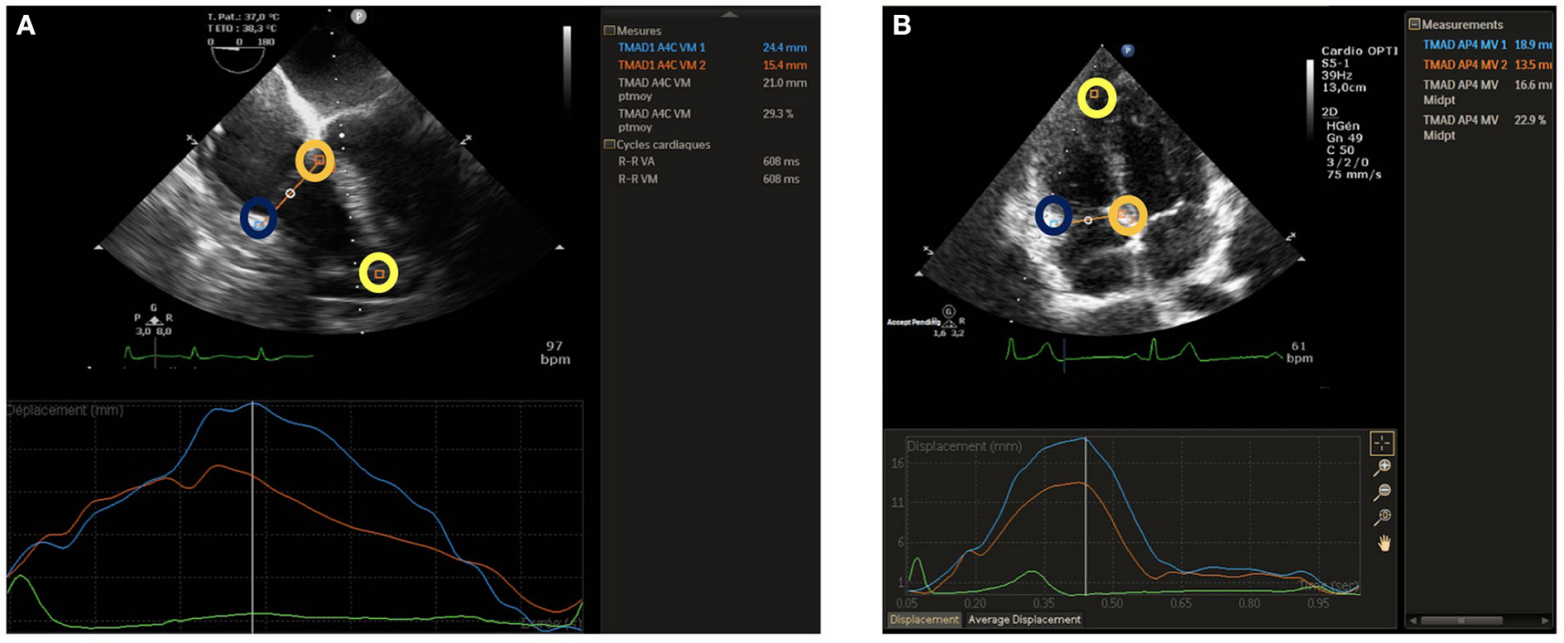
TEE RV-LSF measurement **(A)** and TTE RV-LSF measurement **(B)**. A lateral point (blue circle) and a septal point (orange circle) were placed at the bottom of the RV free wall and the bottom of the interventricular septum. A third point was placed at the apex (yellow circle). TAD lateral, septal, and RV longitudinal shortening fraction (RV-LSF) values were automatically displayed in percentage. The mid-annular point is automated and selected by the software.

RV-LSF was measured using dedicated software (Automated Cardiac Motion Quantification, QLAB version 15.0, Philips Medical Systems, Andover, MA, USA). For RV-LSF analysis, three regions of interest (ROI) were used to initialize the first diastolic frame in a mild-esophageal four-chamber (ME-4CH) view (Figure 1A, Supplementary Video 1) for the TEE procedure and in an RV-focused apical four-chamber view for the TTE procedure (Figure 1B, Supplementary Video 2). These ROI were placed 1) on the tricuspid annulus at the insertion of the anterior tricuspid valve leaflet (RV free wall), 2) on the tricuspid annulus at the insertion of the septal leaflet, and 3) on the RV apex. The software automatically tracked and calculated three parameters: (1) the displacement between the RV free wall and the RV apex (TAD_lat_), (2) the displacement between the interventricular septum and the RV apex (TAD_sep_), and (3) the RV-LSF. RV-LSF was calculated as the maximum end-systolic displacement (LES) of the mid-annular point from the measured annular motion and is expressed as a percentage of the end-diastolic RV longitudinal dimension (LED): 100 × (LED–LES)/LED. The software automatically selected the mid-annular point.

RV-LSF was analyzed in a single beat, and the reported value was the average of 3 measurements. All TTE and TEE RV-LSF measurements were analyzed offline (separately and randomly) by an expert sonographer 2 weeks after the inclusion period.

### RV systolic conventional parameters

In TTE, conventional RV systolic parameters were measured according to international guidelines: tricuspid annular plane systolic excursion (TAPSE) was measured using M-mode with a cursor placed at the junction of the lateral tricuspid leaflet and the RV free wall. RV-S' wave was measured in the apical four-chamber view using Doppler tissue imaging mode. RV systolic and diastolic areas were measured in the apical four-chamber view in 2D mode. RV-fractional area change was calculated by subtracting the end-systolic area from the end-diastolic area and dividing this value by the end-diastolic area. The following variables were recorded: age, gender, body weight, personal medical history, logistic EuroSCORE II, type of cardiac surgery, preoperative plasmatic creatinine, and hemoglobin.

### Statistical analysis

Continuous variables were expressed as mean and 95% confidence interval or standard deviation. Categorical variables are presented as absolute numbers and percentages. The correlation between TTE_RV − LSF_ and TEE_RV − LSF_ measurements was quantified using Pearson's coefficient. Bland-Altman (BA) analysis was performed to assess the level of agreement between RV-LSF_TTE_ and RV-LSF_TEE._

#### Sample size calculation

Assuming a common standard deviation equal to 5 points for each of the RV-LSF values (TTE and TEE), the standard deviation of the difference between the two measures is estimated to be 3.87 if a correlation coefficient (ρ) of 0.7 is assumed between the two RV-LSF measures. Thus, according to Bland and Altman (16), at least 90 evaluable patients would be required to estimate the limits of agreement (LOA) with an accuracy equal to 1 point in RV-LSF. We also performed Bland and Altman analysis to evaluate the levels of agreement between TAD_lat − TTE_, TAD_lat − TEE_, TAD_sep − TTE_, and TAD_sep − TEE._

*Limits of clinical relevance for RV-LSF*: given that the mean RV-LSF_TTE_ from healthy volunteers was 25.6 ± 4.8% (13), we expect a clinically insignificant difference between RV-LSF_TTE_ and RV-LSF_TEE_ to be 5 % (clinical LOA was −5 to 5%). The threshold for statistical significance was set to *p* < 0.05. To analyze the discrepancy between the different measures, we performed a Spearman correlation and a calculation of the intra-class coefficient (ICC). All statistical analyses were performed with R software (version 4.0.4).

## Results

**Figure 2:**
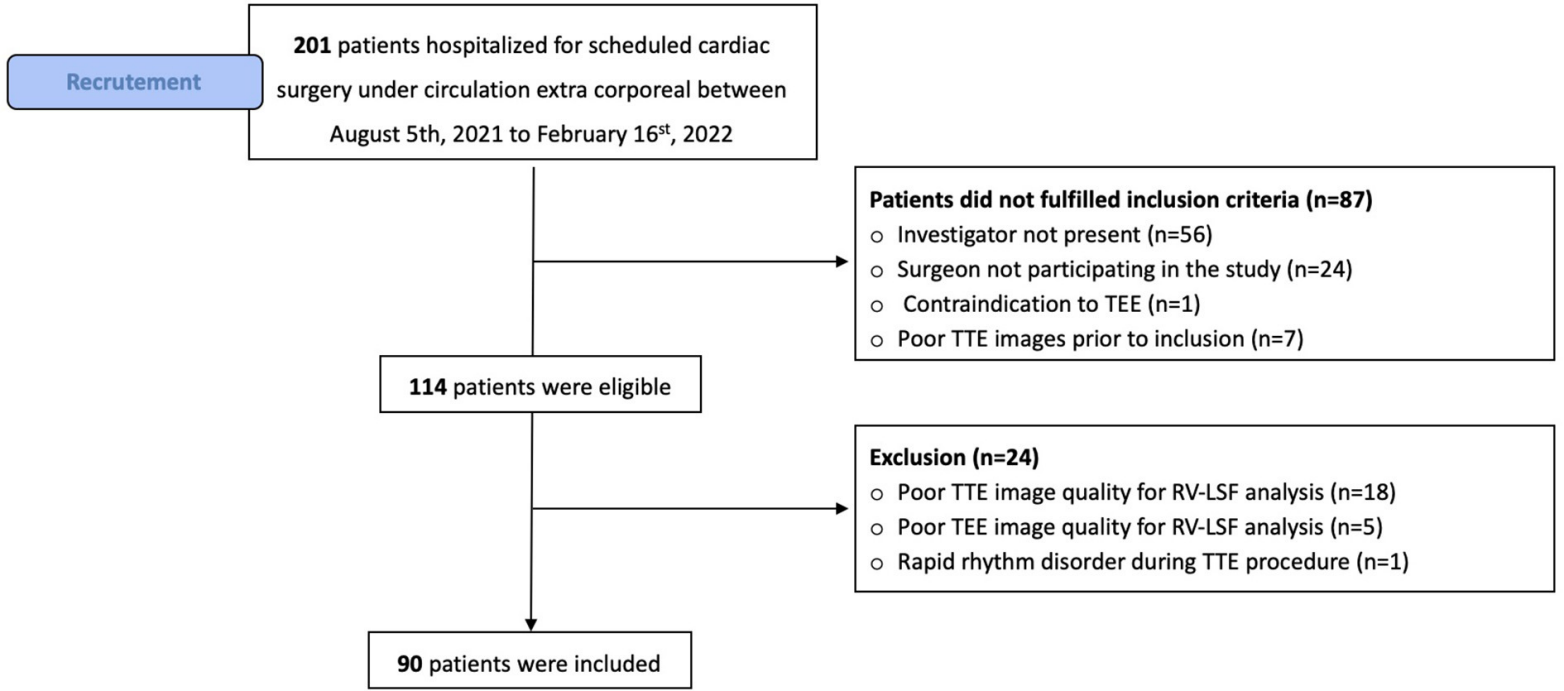
Flow chart of the study.

From August 2021 to February 2022, 201 consecutive patients were hospitalized for scheduled cardiac surgery under cardiopulmonary bypass. Among the 114 patients who met the inclusion criteria, 90 patients were included, and 24 patients were finally excluded: 18 patients for poor TTE image quality, five patients due to a failed ROI placement, and one for rapid rhythm disorder (see Figure 2, Flow chart). Demographic and preoperative data were summarized in Table 1. Among the 90 patients, 82% (*n* = 74/90) were men with an average age of 63 ± 11 years. The average logistic EuroSCORE II was 4.3 ± 4, and valve repair/replacement was the main cardiac surgical procedure (*n* = 33/90, 37%).

**Table 1:** Demographics and echocardiographic data of the population.

**Variables**	**Overall population (*n* = 90)**
Age (years)	63 ± 11
BMI (kg.m^−2^)	27.3 ± 5.8
Male gender *(n %)*	74 (82)
**Medical history**, ***n*** **(%)**	
**Angina severity according to CCS (*****n*** = **32)**	
1	7 (22)
2	7 (22)
3	12 (37)
4	6 (18)
Myocardial infarction	5 (6)
Peripheral vascular disease	11 (12)
Hypertension	53 (59)
Smoking	21 (23)
Diabetes mellitus	19 (21)
Dyslipidemia	57 (63)
Chronic renal disease	6 (6)
Stroke	11 (12)
Atrial fibrillation	20 (22)
Chronic obstructive pulmonary disease	8 (9)
Logistic EuroSCORE (%)	4.3 ± 4
Hemoglobin (g/dl) Creatinine (μmol/l)	14.1 ± 4.7 95 ± 60.9
**Preoperative TTE**	
Left ventricular ejection fraction (%)	59.1 ± 11.2
TAPSE (mm)	21.2 ± 4.8
RV-S' (cm.s^−1^)	12.11 ± 5.6
RV-FAC (%)	47.1 ± 8.2
**Cardiac surgery procedure**, ***n*** **(%)**	
Valve repair/replacement	33 (37)
CABG	26 (29)
Combined	26 (29)
Others	5 (6)
**RV 2D-STE parameter in TTE**	
RV-LSF (%)	20.4 ± 4.3
TAD_lat_ (mm)	20.3 ± 4.8
TAD_sep_ (mm)	11.5 ± 3.1
• RV-LSF (%)	21.1 ± 4.1
• TAD_lat_ (mm)	17.8 ± 4.4
• TAD_sep_ (mm)	9.4 ± 3.1

Data are expressed as mean (standard deviation) and count (%).2D-STE, bi-dimensional speckle tracking echocardiography; BMI, body mass index; CABG, coronary artery bypass graft; CCS, Canadian cardiovascular society; RV, right ventricle; RV-FAC, right ventricle fractional area change; RV-LSF, right ventricular longitudinal shortening fraction; TAPSE, tricuspid annular plane systolic excursion; TAD_lat_, tricuspid annular displacement of the lateral portio; TAD_sep_, tricuspid annular displacement of the septal portion; TEE, transoesophageal echocardiography; TTE, transthoracic echocardiography.

### TTE and TEE RV-LSF measurement

**Figure 3:**
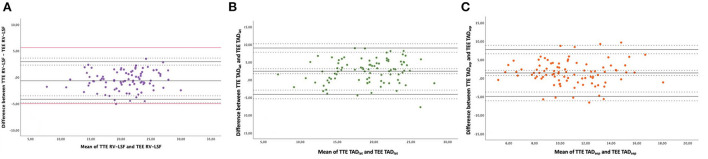
Bland-Altman plots between TTE and TEE measurements with **(A)** TTE RV-LSF vs. TEE RV-LSF. **(B)** TTE TADlat vs. TEE TADlat. **(C)** TTE TADsep vs. TEE TADsep. This plot displays a scatter diagram of the difference between the two techniques' measurements plotted against the average of the two technique's measurements. The black plain line represents the mean of the difference (= bias) between the two ultrasound methods. The other plain lines represent the upper and lower limits of agreement. Dotted black horizontal lines represent the 95% CI interval for the bias and the limits of agreement. The red line represents the determined pertinent clinical agreement (−5; +5%) of the RV-LSF measurement.

**Figure 4:**
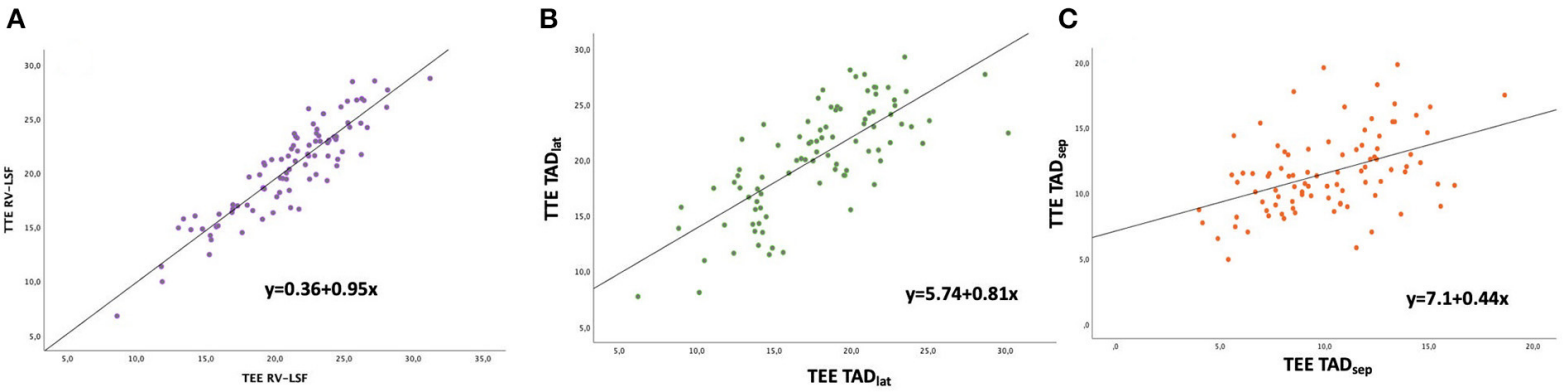
Scatter plots of TTE as a function of TEE measurements of **(A)** RV-LSF **(B)** TAD lateral, and **(C)** TAD septal.

The mean preoperative RV-LSF_TTE_ was 20.4 ± 4.3% and 21.1 ± 4.1% for RV-LSF_TEE_ measurements. Bland-Altman analysis showed an excellent agreement between RV-LSF_TTE_ and RV-LSF_TEE_ measurements. The bias between the two methods was −0.61%, with LOA ranging from −4.18 to 2.97% (Table 2). Figure 3A showed that 95% of RV-LSF measurements fell within the LOA (−4.18 to 2.97 %) and, therefore, within the clinical relevance limits (−5 to 5%) that we had initially determined. Linear regression analysis showed that there was a strong positive correlation between RV-LSF_TTE_ and RV-LSF_TEE_ with a Pearson linear correlation coefficient of 0.91 (CI95% = [0.87–0.94]; *P* < 0.001) and with an excellent model fit (y = 0.36 + 0.95x, r = 0.82, Figure 4A).

**Table 2:** Correlation coefficient and difference between RV-LSF, TADsep, and TADlat measurement in TTE and TTE.

	**TTE _RV − LSF_ vs. TEE _RV − LSF_ (%)**	**TTE _TADsep_ vs. TEE _TADsep_ (mm)**	**TTE_TADlat_ vs. TEE_TADlat_ (mm)**
Difference between measurement	−0,61	1.42	2.44
Lower limit of agreement	−4.18	−4.91	−4.11
Upper limit of agreement	2.97	7.76	8.99
Pearson correlation coefficient [95% CI]	r = 0.91 [0.87–0.94]; *p* < 0.001	r = 0.56 [0.40–0.69]; *p* < 0.001	r = 0.74 [0.63–0.82]; *p* < 0.001
Regression line equation	y = 0.36+0.95x	y = 7,1+0.44x	y = 5.74+0,81x

Data are expressed as numbers. CI, confidence interval; RV-LSF, right ventricular longitudinal shortening fraction; TAD_lat_, tricuspid annular displacement of the lateral portion; TAD_sep_, tricuspid annular displacement of the septal portion; TEE, transoesophageal echocardiography; TTE, transthoracic echocardiography.

### TTE and TEE for TAD_sep_ and TAD_lat_ measurement

The mean preoperative TAD_lat − TTE_ was 20.3 ± 4.8 mm and 17.8±4.4 mm for TAD_lat − TEE_ measurement. Figure 3B showed a reasonable agreement between the two echocardiographic methods for TAD_lat_ measurement with a bias of 2.44 mm and LOA ranging from −4.11 to 8.99 mm (outside the range for clinical agreement). The 2.44 mm bias corresponded to 12.8% relative bias compared to the overall TAD_lat_ measurements with LOA from −21.6 to 47.3%. A good correlation was found between TAD_lat − TTE_ and TAD_lat − TEE_ (r = 0.74, CI95% = [0.63–0.82], *P* < 0.001, Figure 4B). For the TTE and TEE TAD_sep_ measurements, Figure 3C showed that TEE underestimated the TAD_sep_ measurement compared to the TAD_sep − TTE_ measurement with a bias of 1.42 mm and LOA ranging from −4.9 to 7.7 mm. The corresponding relative bias of TAD_sep_ was 13.1% with LOA from −45.2 to 71.0% and moderate correlation between two TAD_sep_ measurements (r = 0.56, CI95% = [0.40–0.69], *P* < 0.001, Figure 4C). We found similar results after calculating the Spearman correlation and intra-class coefficients (Appendix Table 1).

## Discussion

The main findings of the present study can be summarized as follows: (1) RV-LSF measurements using TTE and TEE techniques were interchangeable, (2) TAD_lat_ exhibited a limited bias and good agreement between the two techniques but outside acceptable clinical agreement, and (3) TEE underestimated TAD_sep_ measurement.

### RV-LSF agreement between TTE and TTE

Clinicians need interchangeable RV parameters between TTE and TEE because an accurate assessment of RV systolic function is of utmost importance for perioperative RV monitoring and precise diagnosis of RV dysfunction during postoperative follow-up. The most used TTE RV systolic parameters were RV-FAC and TAPSE, which are assumed to apply to TEE. In TEE, TAPSE measurement is challenging due to a problematic M-mode alignment. Hence, modified methods were proposed for assessing tricuspid systolic excursion with controversial results (7). RV-FAC measurement is limited by the endocardial border definition and its poor reproducibility (8). Despite significant disagreement between TTE and TEE measures (7, 8), RV systolic parameters are often used interchangeably in daily practice.

In this study, we demonstrated that RV-LSF measurements were interchangeable between TTE and TEE. The graphical Bland-Altman analysis showed a limited bias and LOA between the two ultrasound techniques. This is the first study to report an excellent agreement between TTE and TEE techniques using a clinical significance agreement (−5 to 5%) for a 2D-RV global systolic function parameter (8). Several factors explain this result: first, RV-LSF is a highly reproducible, repeatable, and accurate 2D-STE parameter (10). Indeed, RV-LSF is measured semi-automatically by the software (10). Second, unlike RV-strain assessment, RV-LSF measurement does not require high-resolution images. Moreover, during the TEE procedure, tricuspid annulus tracking is less affected by acoustic shadowing than RV-free wall myocardium (needed for strain analysis) (7). Hence, RV-LSF might be helpful in clinical follow-up from admission to the post-operative setting.

### TTE and TEE measurement of TAD lateral and TAD septal

This is the first study that reported data about TAD_lat_ and TAD_sep_ measurement interchangeability. We found that TTE and TEE TAD_sep_ measurements were poorly correlated (r = 0.5) and not interchangeable. We observed a good correlation (r = 0.7) between TTE and TEE TAD_lat_ measurements. However, the graphical Bland-Altman analysis showed that both TAD_lat_ measurement were underestimated, and the LOA between the two methods were too broad for reasonable clinical interchangeability. The underestimation of the TEE measurement was probably because the TEE ME-4CH view may not represent the true long axis of the interventricular septum and causes a “foreshortening” view. Foreshortening view is a frequent problem in 2D echocardiography examinations. It occurs when the ultrasound beam does not cut through the true apex, leading to geometric distortion of the image. Therefore, the long axis of the ventricle appears shorter. Previous studies on LV function identified similar findings about underestimation of volumes due to foreshortening of the transesophageal imaging plane (17). The impact of the foreshortening view on RV-LSF measurement was probably limited because RV-LSF is a length ratio and because the underestimation of TAD_sep_ and TAD_lateral_ values, on which RV-LSF value depends, is relatively homogeneous (close to 12% for both).

Besides, as in our study, these results are probably related to using the RV apex as the reference point. Selection of RV apex can be challenging because TEE only partially reveals the apical portion of the RV, which is truncated or shortened; this leads to underestimating the measured parameters. The use of 3D echocardiography for assessing the RV systolic function is the best approach to avoid a foreshortening view and an underestimation of ventricle size or volumes (18).

Our results were close to that of other studies which assess the interchangeability between the longitudinal displacement analysis of the lateral portion of the tricuspid annular and TAPSE. Markin et al. compared TTE TAPSE by M-mode and TEE TAPSE by speckle tracking in 84 patients. They found that TAPSE by M-mode was correlated with TAPSE by speckle tracking in the ME-4CH view (Pearson r = 0.62), but they did not analyze the agreement between the two methods. In the study by Mauermann et al., TAD lateral (named speckle tracking TAPSE) was assessed in TEE and compared to TTE TAPSE. The authors found a significant correlation (r = 0.59) but with large LOA (−9.4 to 8.4 mm) (7).

## Strengths and limitations

This study had several strengths. First, this study prospectively acquired TTE and TEE images under identical clinical situations in mechanically ventilated patients under general anesthesia. Secondly, we performed a sample size calculation to assess clinical agreement between the two techniques to avoid underpowered analysis. Nevertheless, this study admits some limitations. First, RV-LSF_TEE_ and RV-LSF_TTE_ were calculated from loops recorded in a supine position, possibly resulting in a foreshortened apical view in TTE. To limit the impact of a foreshortening apical view on RV long-axis measurement due to supine position, we measured RV-LSF in an RV-focused apical four-chamber view as recommended (3). Second, we did not evaluate the inter-observer reproducibility for TTE and TEE for RV-LSF measurement. Indeed, because both ultrasound procedures were performed just before the surgical draping of the patient, we chose to shorten the duration of image acquisition to avoid any extensive delay before starting the surgical procedure. Third, TEE and TTE measurements were acquired by an echocardiography expert (level III competence according to the EACVI definition) (19) because the main issue is related to the imaging window. The ROI positioning on the lateral part of the tricuspid annular or the RV-apex may be limited because the sector window is too narrow and requires an optimal view. We believe that RV-LSF measurement should be performed by a physician with advanced training in TTE. Fourth, we failed to measure RV-LSF_TEE_ in four patients with large aortic root. Hence, the use of RV-LSF appears to be limited for monitoring RV systolic function in aortic root surgery. Finally, as with many 2D-STE parameters, the software version is a potential limitation. It is possible that RV-LSF values measured by the Philips QLAB version 15.0 may not reflect results from another version of the same software (20, 21).

## Conclusion

In this study, we showed that RV-LSF_TTE_ and RV-LSF_TEE_ measured in the operating room for patients undergoing cardiac surgery exhibited excellent clinical agreement, and thus were interchangeable. RV-LSF could be helpful in assessing RV systolic function during and after high-risk surgery.

## Data availability statement

The raw data supporting the conclusions of this article will be made available by the authors, without undue reservation.

## Ethics statement

The studies involving human participants were reviewed and approved by Comité de Protection des Personnes Ile de France VIII, ID-RCB 2021_A000908-33. The patients/participants provided their written informed consent to participate in this study.

## Author contributions

Study conception and manuscript drafting: CB, OA-A, DM, and YM. Clinical data collection: PV, FL, CD, PH, BA, GT, and TC. Statistical analysis: BR and DM. Manuscript revision: CB, HD, and YM. All authors approved the final version of the manuscript.

## Appendix

**Table A1:** Spearman correlation coefficient and ICC between RV-LSF, TAD_sep_, and TAD_lat_ measurement in TTE and TTE.

	**Spearman correlation coefficient**	**ICC**
RV-LSF_TTE_ vs. RV-LSF_TEE_	0.89 [0.82–0.93]	0.91 [0.86–0.96]
TAD_lat − TTE_ vs. TAD_lat − TEE_	0.74 [0.63–0.82]	0.74 [0.63–0.82]
TAD_sep − TTE_ vs. TAD_sep − TEE_	0.46 [0.25–0.62]	0.44 [0.26–0.59]

Data are expressed in numbers and 95% confidence interval [-]. ICC, interclass coefficient; RV-LSF, right ventricular longitudinal shortening fraction; TAD_lat_, tricuspid annular displacement of the lateral portion; TAD_sep_, tricuspid annular displacement of the septal portion; TEE, transoesophageal echocardiography; TTE, transthoracic echocardiography.
